# Impact of Radiometal Chelates on In Vivo Visualization of Immune Checkpoint Protein Using Radiolabeled Affibody Molecules

**DOI:** 10.1021/acsptsci.4c00539

**Published:** 2025-02-19

**Authors:** Vladimir Tolmachev, Eleftherios Papalanis, Ekaterina A. Bezverkhniaia, Alia Hani Rosly, Anzhelika Vorobyeva, Anna Orlova, Matilda Carlqvist, Fredrik Y. Frejd, Maryam Oroujeni

**Affiliations:** † Department of Immunology, Genetics and Pathology, Uppsala University, 751 85 Uppsala, Sweden; ‡ Department of Medicinal Chemistry, Uppsala University, 751 83 Uppsala, Sweden; § Affibody AB, 171 65 Solna, Sweden

**Keywords:** B7−H3, Affibody molecule, indium-111(^111^In), technetium-99m (^99m^Tc), residualizing, nonresidualizing, DOTA chelator, SKOV-3 xenograft, SPECT/CT imaging

## Abstract

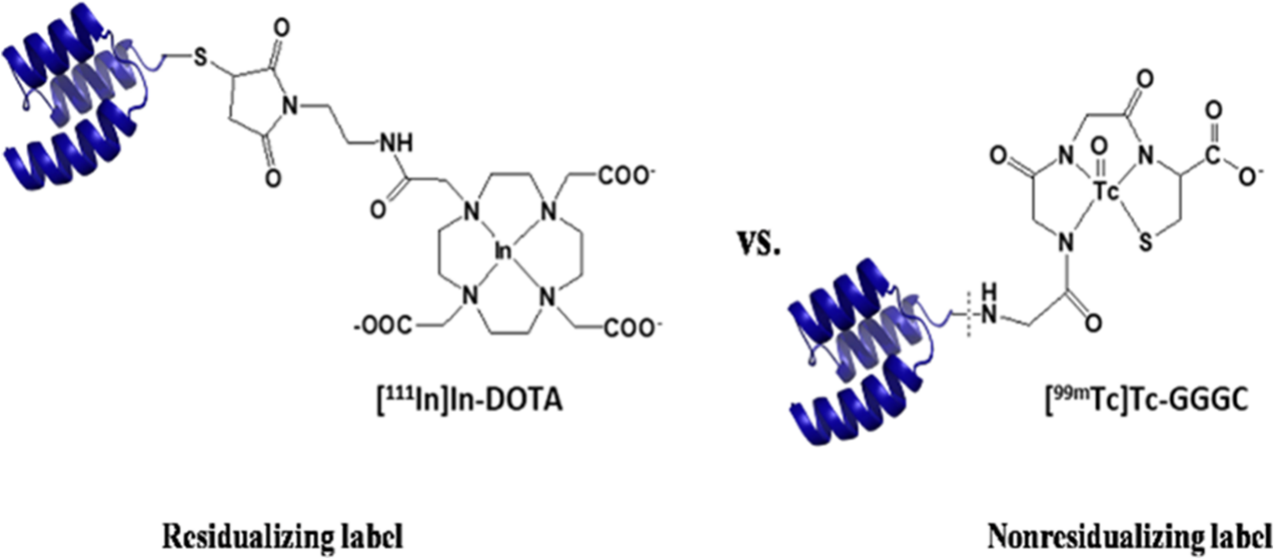

The immune checkpoint protein B7–H3 (CD276) is overexpressed in various cancers and is an attractive target for the treatment of malignant tumors. Radionuclide molecular imaging of B7–H3 expression using engineered scaffold proteins such as Affibody molecules is a promising strategy for the selection of potential responders to B7–H3-targeted therapy. Feasibility of B7–H3 imaging was demonstrated using two ^99m^Tc-labeled probes, AC12 and an affinity-matured SYNT179 using a [^99m^Tc]Tc-GGGC label. This study aimed to evaluate whether the use of a residualizing ^111^In-based label provides better imaging contrast compared with a nonresidualizing label. To do that, SYNT179 and AC12-GGGC Affibody molecules were labeled with ^111^In using (4,10-bis-carboxymethyl-7-{[2-(2,5-dioxo-3-thioxo-pyrrolidin-1-yl)-ethylcarbamoyl]-methyl}-1,4,7,10-tetraaza-cyclododec-1-yl)-acetic acid (maleimide-DOTA) chelator, site-specifically coupled to the C-terminus of Affibody molecules. The binding affinities of the ^111^In-labeled conjugates to B7–H3-expressing living cells were higher compared with the affinities of the ^99m^Tc-labeled variants. In mice with B7–H3-expressing xenografts, the tumor uptake of ^111^In-labeled proteins (3.6 ± 0.3 and 1.8 ± 0.5%ID/g for [^111^In]In-SYNT179-DOTA and [^111^In]In-AC12-DOTA, respectively) was significantly (*p* < 0.05, ANOVA) higher than those for ^99m^Tc-labeled counterparts (1.6 ± 0.2%ID/g and 0.8 ± 0.2%ID/g for [^99m^Tc]Tc-SYNT179 and [^99m^Tc]Tc-AC12-GGGC, respectively). The best variant, [^111^In]In-SYNT179-DOTA, provided a tumor-to-blood ratio of 31.1 ± 2.9, which was twice higher than that for [^99m^Tc]Tc-SYNT179 and 7-fold higher than that for [^99m^Tc]Tc-AC12-GGGC. Both ^111^In-labeled Affibody molecules had higher renal retention compared with ^99m^Tc-labeled ones, but the hepatobiliary excretion of ^111^In-labeled proteins was appreciably lower, potentially improving the imaging of abdominal metastases. Overall, [^111^In]In-SYNT179-DOTA is the most promising tracer for visualization of B7–H3 expression.

Radionuclide molecular imaging uses the molecular recognition of targets that are highly expressed in tumor sites compared to normal tissues. The promising clinical responses to antibody-based therapies have prompted the identification of clinically relevant cell membrane proteins, which can serve as targets in tumors. One of the emerging molecular targets for radionuclide diagnosis and therapy is B7–H3, also known as CD276 or B7RP-2. This transmembrane glycoprotein, comprising 316 amino acids, belongs to the B7 ligand family of immune checkpoint proteins.^1^

The exact function of B7–H3 is still not fully understood, and it appears to play complex roles in immune regulation. Some studies suggest that B7–H3 has a dual role, which includes costimulatory and coinhibitory effects.^2^ B7–H3 has immunologic functions such as suppression of proliferation, activation, and cytotoxicity of T-cells. Moreover, this immune checkpoint protein has nonimmunologic functions such as promotion of cancer cell invasion and migration, enhancement of cancer cell proliferation and metabolism, apoptosis evasion, and stimulation of tumor angiogenesis.^3^ It is involved in modulating the activity of immune cells. B7–H3 is expressed on the surface of certain types of immune and nonimmune cells and various tumor cells. The expression of B7–H3 can be upregulated in response to different stimuli, including inflammation and cancer. Importantly, B7–H3 is an attractive target for antibody-based immunotherapy.^4,5^ B7–H3 is expressed at high frequency in numerous cancer types (60% of all cancers) while exhibiting a limited expression at low levels in normal tissues.^6,7^ There is compelling evidence that B7–H3 overexpression in certain types of cancer correlates with poor prognosis, limited choice of treatments, chemoresistance, and an increased risk of cancer recurrence and metastasis.^8^ It has also been found that there is a corrolation between the serum level of B7–H3 and the histopathological findings in tumors, making B7–H3 a prognostic biomarker.^8^ One of the issues in the use of B7–H3 as a clinical biomarker or molecular therapy target is the heterogeneity of its expression, which was observed in several types of malignant tumors, such as in neuroendocrine pancreatic, sarcomatoid pulmonary, and colorectal malignancies.^9−11^ It was stated that some factors, such as expression heterogeneity and variation, have to be carefully considered in translating B7–H3-targeted therapies.^12^

Biopsy sampling, as a widely used method to determine the expression level of receptors in tumors, does not provide comprehensive information about overall target expression. The use of this method is associated with several limitations, such as the restricted number of samples that can be taken from patients, a high degree of heterogeneity, different expression levels in primary tumors and metastases, and changes in target expression during therapy. Radionuclide molecular imaging of B7–H3 is a potentially facile and noninvasive approach to determine the levels of its expression in tumors. Selection of an optimal format for an imaging agent is critical to providing sufficient sensitivity and specificity for such diagnostics. Several anti-B7–H3-targeting agents, such as monoclonal antibodies (mAbs), bispecific antibodies, antibody–drug conjugates, and radioimmunotherapy agents, have shown promising antitumor activities, acceptable safety in preclinical models, and some phase I clinical trials in patients.^3,13^ However, a slow extravasation rate into the tumor and slow clearance from blood circulation and nontargeted organs are the main disadvantages of the use of mAbs for imaging, resulting in low imaging contrast at the day of injection and a relatively high dose burden to the patients.

Affibody molecule, an engineered nonimmunoglobulin scaffold protein, is a type of small targeting agent with a size of 6–7 kDa (approximately 25 times smaller than intact monoclonal antibodies).^14^ Affibody molecules offer various attractive features, and the most important advantageous characteristics are a high extravasation rate into tumors and rapid clearance from the bloodstream and normal organs and tissues due to their small size, resulting in an enhanced imaging contrast in a few hours after administration.^15,16^ Importantly, the folding of Affibody molecules into a trihelical structure occurs in physiologic conditions spontaneously, without the involvement of disulfide bonds.^15^ Therefore, the Affibody scaffold does not contain cysteines. Simple gene engineering enables the incorporation of cysteine in a desirable position in the scaffold, creating a unique thiol group. The use of a sulfhydryl-directed conjugation (e.g., maleimide-mediated) permits a site-specific coupling of chelators or other prosthetic groups to Affibody molecules.^15,16^ In the previous studies, this process was optimized, providing homogeneous Affibody–chelator conjugates with reproducible biodistribution. Affibody molecules can also be site-specifically labeled with ^99m^Tc, ^186^Re, or ^188^Re using a peptide-based N_3_S chelator formed by a thiol group of the C-terminal cysteine and three nitrogens from amide groups.^15,16^ The potential of Affibody-based agents for ultrasound, photoacoustic, and fluorescence imaging of B7–H3 has been demonstrated recently in preclinical breast cancer models.^17,18^ We have demonstrated the feasibility of preclinical molecular imaging of B7–H3-expressing tumors a few hours after injection using the ^99m^Tc-labeled Affibody molecule.^19^

One of the challenges for imaging is the relatively low expression level of B7–H3 in tumors, which is often around 45,000–60,000 molecules per cell.^19^ The accumulation and retention of radioactivity in tumors with such low expression levels depend on several factors, including an interplay between the internalization rate and affinity. Slow internalization after binding to cancer cells is a common characteristic of many engineered scaffold proteins, including Affibody molecules,^20−22^ albumin-derived affinity proteins (ADAPTs),^23^ and designed ankyrin repeat proteins (DARPins).^24,25^ Due to the slow internalization of Affibody molecules, a good retention of activity in tumors should strongly depend on the high affinity of the targeting protein and to a lesser extent on the residualizing properties of the radiolabel. The results of a recent preclinical study demonstrated that affinity maturation improved biodistribution and imaging properties of Affibody molecules used for imaging of B7–H3-expressing tumors.^26^ In our previous studies, a C-terminal N_3_S chelator formed by amide nitrogens and a thiol group from cysteine in triglycine-cysteine (GGGC) was used for labeling of B7–H3-targeting Affibody molecules with ^99m^Tc (Figure 1).^19,26^ These and some other studies have demonstrated that [^99m^Tc]Tc-GGGC behaves as a nonresidualizing label showing, e.g., a rapid washout from kidneys. However, tumor-to-blood ratios are typically higher for Affibody molecules carrying residualizing labels.^16^ The blood-borne activity may impact an imaging contrast.

**Figure 1:**
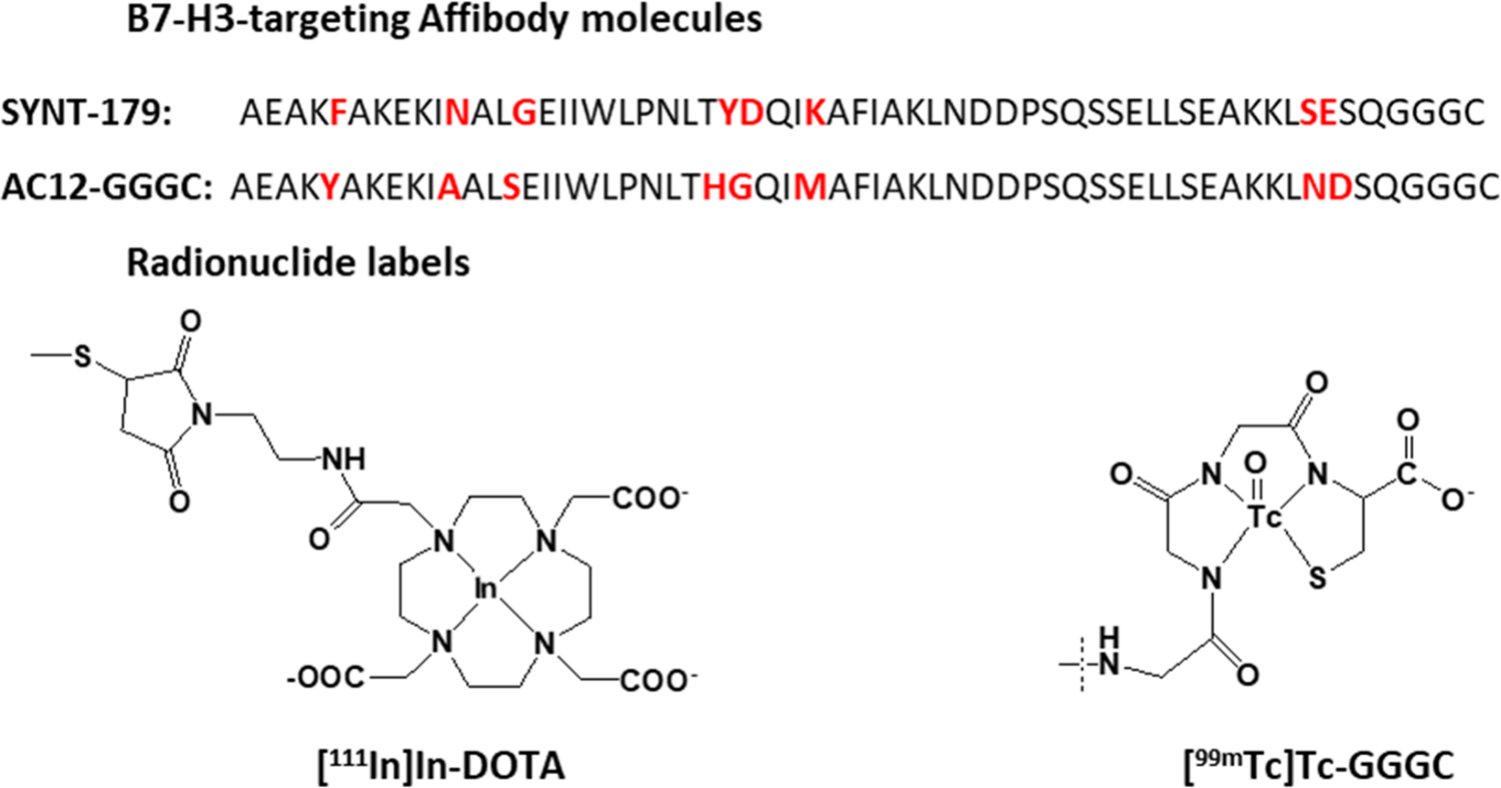
Sequences of AC12 and SYNT179. Amino acids differing in AC12 and SYNT179 are marked in red. Schematic structures of [^111^In]In-DOTA and [^99m^Tc]Tc-GGGC labels.

In this study, we hypothesized that the use of a residualizing ^111^In-label would influence biodistribution and the tumor-to-organ ratios (imaging contrast) of the B7–H3-targeting Affibody molecules compared to the nonresidualizing ^99m^Tc-labeled counterparts.

To test our hypothesis, we used B7–H3-targeting Affibody molecules AC12 and affinity-matured SYNT179 (Figure 1). A complex of ^111^In with a DOTA chelator was used as a residualizing label. For site-specific labeling, the maleimido-monoamide-DOTA was conjugated to the C-termini of these Affibody molecules. The C-terminal GGGC sequence was used to label these targeting proteins with ^99m^Tc to provide counterparts with a nonresidualizing label for in vivo comparison. It should be noted that the conjugation/labeling was site-specific, and we had only one chelating site per Affibody molecule. In vitro experiments (to test specificity, affinity, and internalization) were performed using B7–H3-expressing cell lines. Biodistribution of the ^111^In- and ^99m^Tc-labeled Affibody molecules was compared in the same batch of mice implanted with B7–H3-expressing SKOV-3. Mice implanted with B7–H3-negative Ramos were used to test specificity of ^111^In-labeled Affibody molecules in vivo. SPECT/CT imaging of ^111^In-labeled Affibody molecules was performed.

Several studies have demonstrated that efficient clearance of metabolites is crucial for achieving optimal imaging contrast and, consequently, maximum sensitivity. A clinical study using HER2-binding Affibody molecules has shown that imaging at the 4 h time point provides superior contrast, particularly for detecting hepatic metastases, compared to imaging at 1 or 2 h post injection.^27^ Therefore, the 4 h time point is highly relevant for clinical translation.

## 1 Results

### 1.1 Production, Purification, and Characterization of Anti-B7–H3 Affibody Molecules

**Figure 2:**
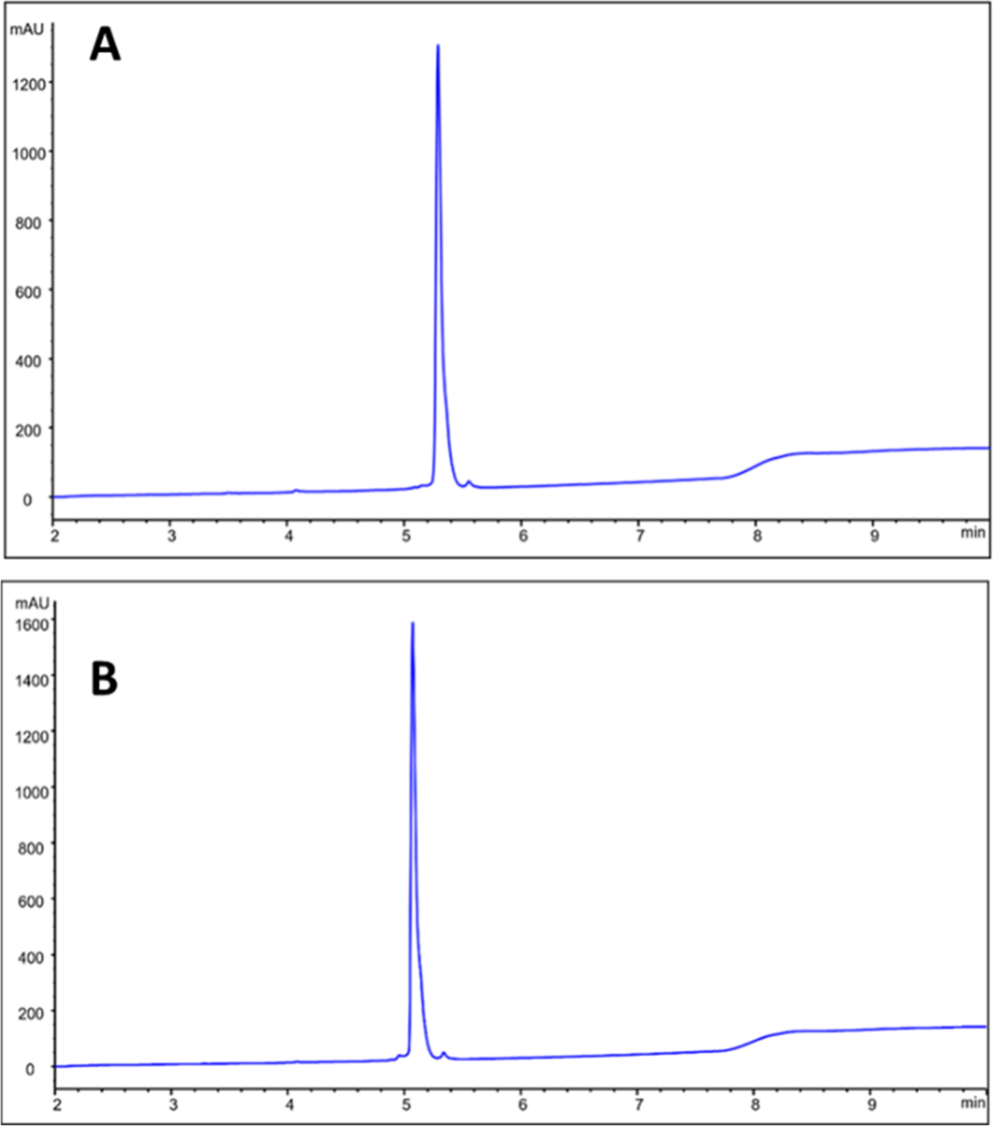
HPLC chromatogram at 220 nm of (A) SYNT179-DOTA (retention time: 5.29 min) and (B) AC12-DOTA (retention time: 5.07 min).

The conjugated binders were characterized by HPLC-MS, and correct masses were confirmed by single quadrupole mass spectrometry (Table 1). The purity of both Affibody molecules was determined to be 96% by integration of the chromatograms (Figure 2).

**Table 1:** Analysis Results from RP-HPLC-MS of DOTA Conjugated to Affibody Molecules

	theoretical mass (Da)	experimentally determined mass (Da)	purity (%)
SYNT179-DOTA	6936.8	6937.1	96
AC12-DOTA	6814.7	6815.0	96

### 1.2 Radiolabeling and In Vitro Stability

Table 2 shows the results of labeling of anti-B7–H3 Affibody molecules with ^111^In. Both variants were successfully labeled with ^111^In. The radioconjugates were purified using size exclusion chromatography with the NAP-5 column, providing radiochemical purities >95%. Both radiolabeled conjugates demonstrated sufficient stability during incubation in PBS for up to 24 h (Table 3).

**Table 2:** ^111^In Labeling of Anti-B7–H3 Affibody Molecules^a^

	radiochemical yield, %	radiochemical yield after EDTA treatment, %	radiochemical purity, %	maximal specific activity, MBq/μg
SYNT179-DOTA	91 ± 3	88 ± 1	99 ± 1	0.5
AC12-DOTA	91 ± 6	90 ± 1	99 ± 1	0.5

a The data are expressed as the mean value from three samples ± SD.

**Table 3:** In Vitro Stability of ^111^In-Labeled Affibody Molecules^a^

	protein-associated activity, %		
	PBS		
	1 h	4 h	24 h
[^111^In]In-SYNT179-DOTA	93 ± 1	93 ± 1	91 ± 1
[^111^In]In-AC12-DOTA	97 ± 1	93 ± 2	92 ± 2

a The stability test was performed in triplicates.

**Figure 3:**
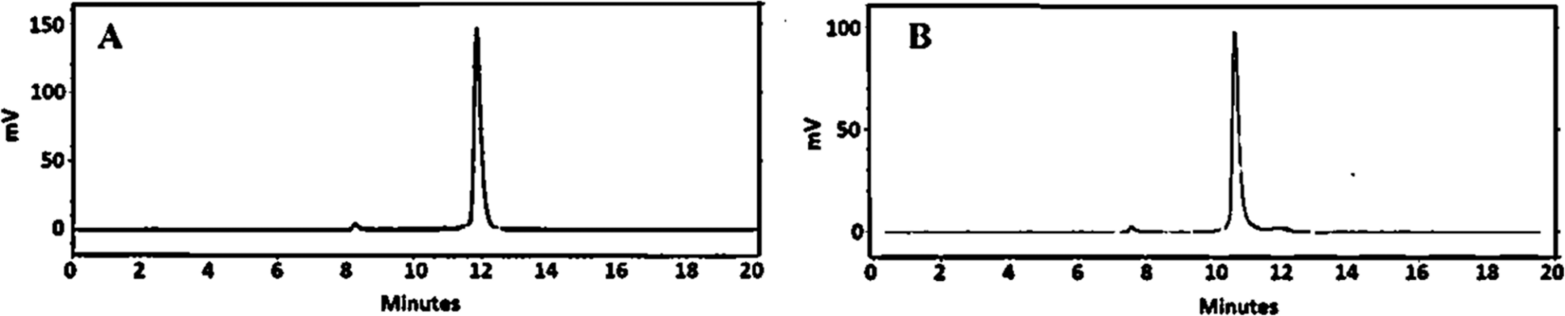
Reversed-phase HPLC radiochromatogram of (A) [^111^In]In-SYNT179-DOTA and (B) [^111^In]In-AC12-DOTA Affibody molecules. The retention times are presented in minutes.

The retention time of ^111^In-labeled conjugates was around 10–12 min (according to radio-HPLC radiochromatogram, Figure 3A,B). A single prominent peak was observed, corresponding to the radiolabeled Affibody molecule.

### 1.3 In Vitro Studies

**Figure 4:**
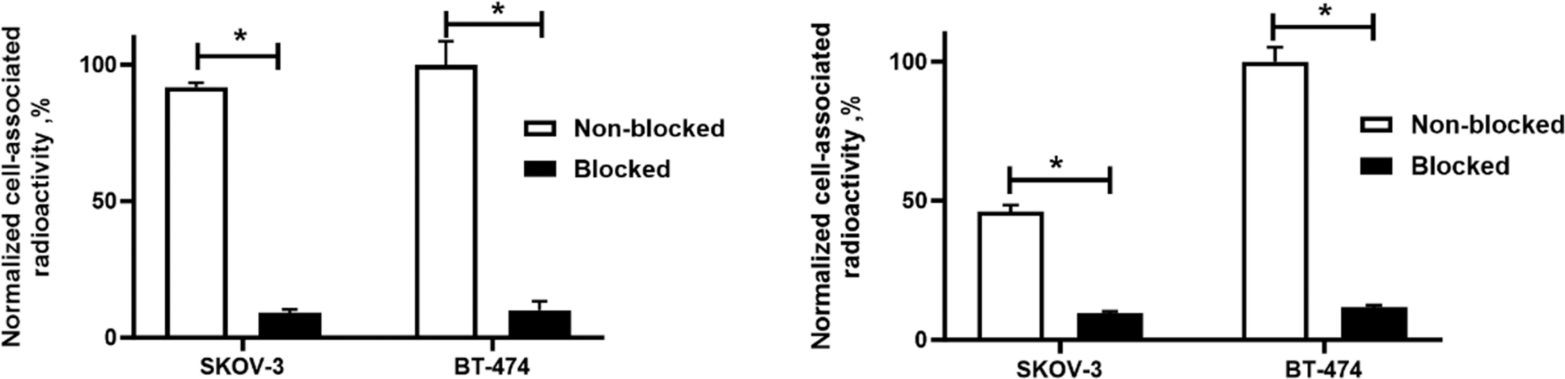
In vitro specificity of (A) [^111^In]In-SYNT179-DOTA and (B) [^111^In]In-AC12-DOTA binding to SKOV-3 and BT-474 cells. Asterisk (*) was used to show significant (*p* < 0.05) differences.

The cell-associated activity was significantly (*p* < 0.05) decreased when the cells were presaturated with the excess amount of nonlabeled anti-B7–H3 Affibody molecules before adding the radiolabeled one (Figure 4), demonstrating that the binding was target-specific.

The results of binding kinetic measurements are presented in Table 4. According to InteractionMap modeling, the best fitting of the kinetics of the [^111^In]In-SYNT179-DOTA and [^111^In]In-AC12-DOTA binding to the SKOV-3 cell line was achieved using a 1:2 model. In agreement with the previous data,^19,26^ both Affibody variants demonstrated two types of interactions with living B7–H3-expressing cells, namely, a high-affinity interaction with low abundance and an interaction with lower affinity but with higher abundance. Furthermore, the most abundant interaction of [^111^In]In-SYNT179-DOTA had higher strength (lower K_D2_ value) compared with the interaction of [^111^In]In-AC12-DOTA. The chemical nature of the label had an impact on the interactions. The use of ^111^In resulted in an increased abundance of the high-affinity interaction compared with the use of ^99m^Tc in the case of SYNT179. In the case of AC12, there was some increase in the strength for both interactions.

**Table 4:** Apparent Equilibrium Dissociation (K_D_) Constants for the Interaction between Labeled Affibody Molecules and SKOV-3 Cells Determined Using an InteractionMap Analysis of the LigandTracer Sensorgrams

	*K*_D1_ (nM)	weight_1_,%	*K*_D2_ (nM)	weight_2_, %
[^111^In]In-SYNT179-DOTA	0.06 ± 0.04	20	13 ± 2	80
[^111^In]In-AC12-DOTA	0.9 ± 0.2	8	50 ± 5	92
[^99m^Tc]Tc-SYNT179^26^	0.028 ± 0.001	12	8.2 ± 0.5	88
[^99m^Tc]Tc-AC12-GGGC^19^	1.9 ± 0.8	23	68.8 ± 7.4	67

**Figure 5:**
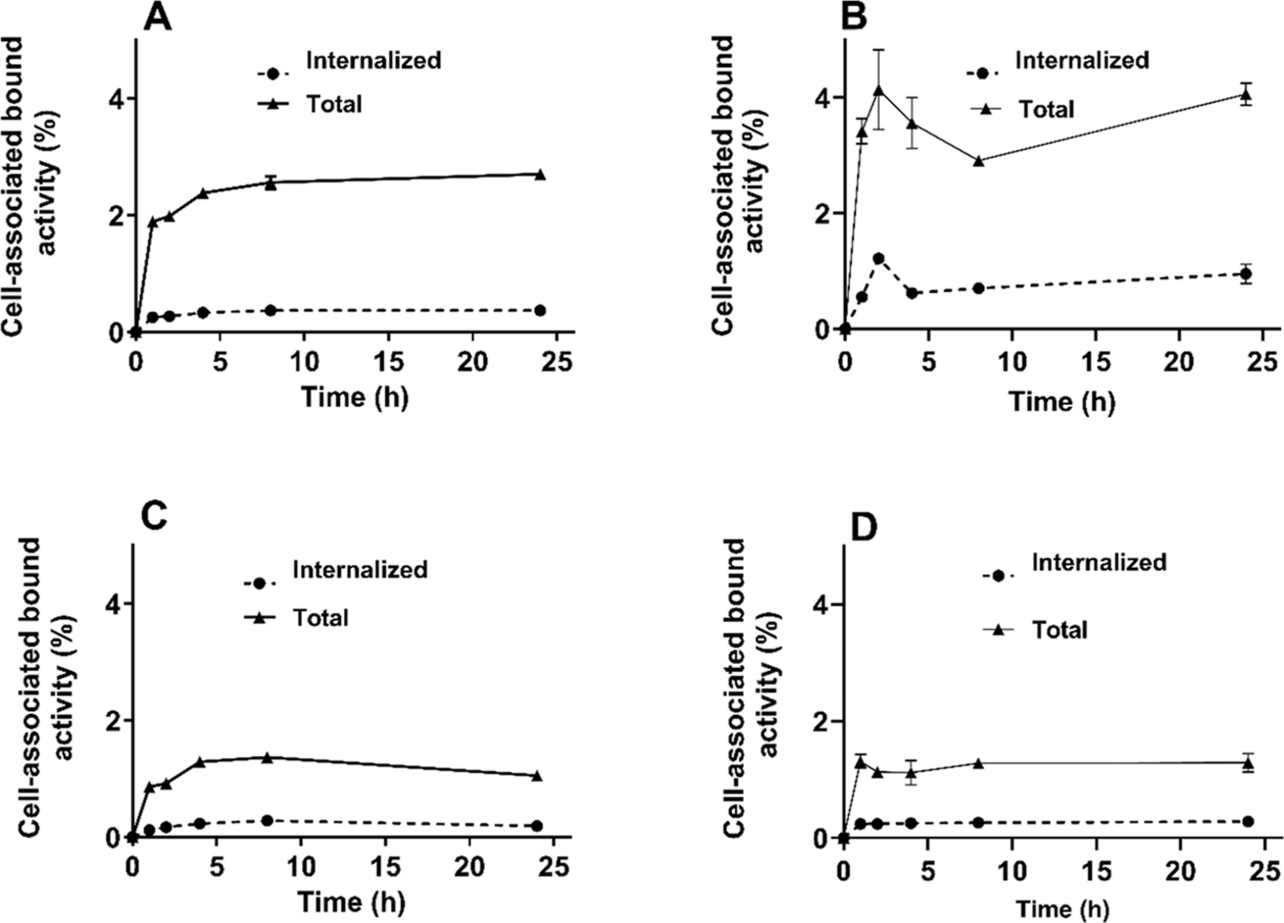
Cellular processing and internalization of (A, B) [^111^In]In-SYNT179-DOTA and (C, D) [^111^In]In-AC12-DOTA on SKOV-3 (A, C) and BT-474 (B, D) cells.

The cellular processing and internalization assay (Figure 5) demonstrated that total cell-associated activity was 2.7 ± 0.1% on SKOV-3 and 3.8 ± 0.6% on BT-474 for [^111^In]In-SYNT179-DOTA and 1.05 ± 0.06% on SKOV-3 and 1.3 ± 0.2% on BT-474 for [^111^In]In-AC12-DOTA after 24 h of incubation. The overall internalization pattern in vitro was similar for both radioconjugates, characterized by a relatively slow internalization rate. However, [^111^In]In-SYNT179-DOTA exhibited higher internalized activity (0.37 ± 0.01% on SKOV-3 and 0.77 ± 0.15% on BT-474) compared to [^111^In]In-AC12-DOTA (0.19 ± 0.03% on SKOV-3 and 0.28 ± 0.05% on BT-474).

### 1.4 In Vivo Studies

**Figure 6:**
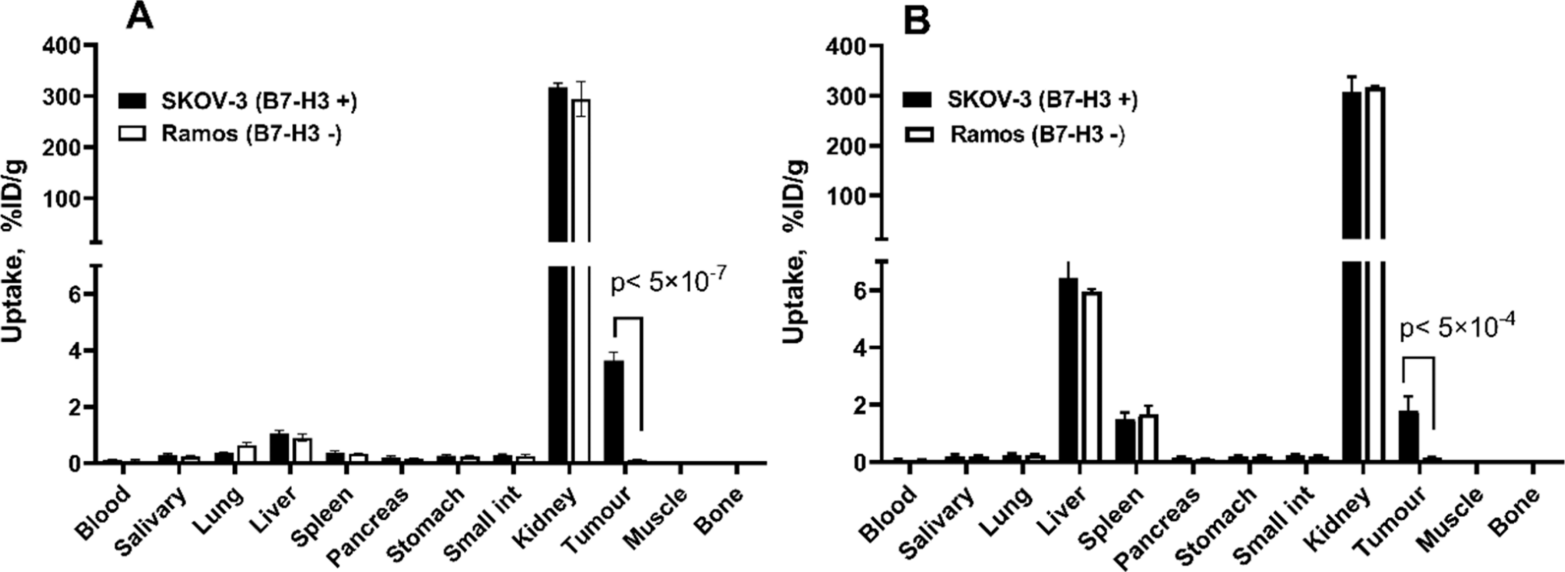
Uptake of (A) [^111^In]In-SYNT179-DOTA and (B) [^111^In]In-AC12-DOTA in SKOV-3 (B7–H3-positive) and Ramos (B7–H3-negative) xenografts 4 h after injection. Data are presented as the percentage of injected dose per gram of tissue (% ID/g) and are averages from four mice ± SD.

Biodistribution measurements showed that the uptake of both ^111^In-labeled conjugates in B7–H3-positive SKOV-3 xenografts was significantly (*p* < 0.005) higher than that in B7–H3-negative Ramos xenografts 4 h after injection (Figure 6). The results demonstrated that the tumor uptake was target-mediated in vivo.

The results of biodistribution of SYNT179-DOTA and AC12-DOTA labeled with ^111^In at 4 and 24 h after injection are presented in Table 5. Biodistribution demonstrated that the blood concentration of [^111^In]In-SYNT179-DOTA (0.12 ± 0.01%ID/g) was significantly (*p* < 0.05) higher than that for [^111^In]In-AC12-DOTA (0.09 ± 0.02%ID/g) at 4 h p.i. The tumor uptake of [^111^In]In-SYNT179-DOTA was 3.63 ± 0.31 and 0.78 ± 0.18%ID/g at 4 and 24 h p.i., respectively, which were significantly (*p* < 0.05) higher than the tumor uptakes for [^111^In]In-AC12-DOTA (1.80 ± 0.49 and 0.37 ± 0.13%ID/g at 4 and 24 h p.i., respectively). [^111^In]In-SYNT179-DOTA showed significantly (*p* < 0.05) lower hepatic uptake at both time points of the study (1.07 ± 0.08 and 0.80 ± 0.02%ID/g at 4 and 24 h p.i., respectively) than the uptake of [^111^In]In-AC12-DOTA (6.43 ± 1.05 and 4.07 ± 0.21 at 4 and 24 h p.i., respectively). A quick washout of activity from blood and almost all organs and tissues over time was observed for both radioconjugates. Also, the uptake of both conjugates in tumors was significantly (*p* < 0.05) lower at 24 h after injection than after 4 h.

**Table 5:** Head-to-Head Biodistribution of ^111^In-Labeled Affibody Molecules in BALB/C nu/nu Mice Bearing SKOV-3 Xenografts 4 and 24 h p.i^a^

	uptake, %ID/g			
	[^111^In]In-SYNT179-DOTA		[^111^In]In-AC12-DOTA	
site	4 h	24 h	4 h	24 h
blood	0.12 ± 0.01^b^^,^^d^	0.019 ± 0.002^b^	0.09 ± 0.02^c^^,^^d^	0.018 ± 0.001^c^
salivary glands	0.28 ± 0.05	0.23 ± 0.04^e^	0.20 ± 0.07^c^	0.11 ± 0.01^c^^,^^e^
lung	0.36 ± 0.02^b^^,^^d^	0.19 ± 0.02^b^^,^^e^	0.25 ± 0.05^c^^,^^d^	0.09 ± 0.03^c^^,^^e^
liver	1.07 ± 0.08^b^^,^^d^	0.80 ± 0.02^b^^,^^e^	6.43 ± 1.05^c^^,^^d^	4.07 ± 0.21^c^^,^^e^
spleen	0.38 ± 0.04^d^	0.37 ± 0.06^e^	1.49 ± 0.24^d^	1.14 ± 0.14^e^
pancreas	0.21 ± 0.04^b^	0.12 ± 0.01^b^^,^^e^	0.15 ± 0.05^c^	0.06 ± 0.02^c^^,^^e^
stomach	0.26 ± 0.03^b^^,^^d^	0.16 ± 0.04^b^	0.21 ± 0.02^c^^,^^d^	0.10 ± 0.03^c^
small intestine	0.29 ± 0.03^b^	0.21 ± 0.04^b^^,^^e^	0.24 ± 0.03^c^	0.11 ± 0.04^c^^,^^e^
kidney	317.06 ± 8.16^b^	270.72 ± 8.43^b^	308.27 ± 30.02	225.57 ± 58.89
tumor	3.63 ± 0.31^b^^,^^d^	0.78 ± 0.18^b^^,^^e^	1.80 ± 0.49^c^^,^^d^	0.37 ± 0.13^c^^,^^e^
muscle	0.04 ± 0.01	0.03 ± 0.01^e^	0.03 ± 0.01	0.02 ± 0.01^e^
bone	0.02 ± 0.01^b^	0.014 ± 0.001^b^	0.018 ± 0.001^c^	0.008 ± 0.005^c^
low GI tract^f^	0.54 ± 0.15	0.27 ± 0.03	0.84 ± 0.24	0.18 ± 0.04
body^f^	3.22 ± 0.07	2.40 ± 0.27	2.59 ± 0.39	1.86 ± 0.67

a The data are presented as the average value (n = 4) ± SD.

b Significant difference (*p* < 0.05): between [^111^In]In-SYNT179-DOTA at 4 and 24 h p.i.

c Significant difference (p < 0.05) between [^111^In]In-AC12-DOTA at 4 and 24 h p.i.

d Significant difference (p < 0.05) between [^111^In]In-SYNT179-DOTA and [^111^In]In-AC12-DOTA at 4 h p.i.

e Significant difference (p < 0.05) between [^111^In]In-SYNT179-DOTA and [^111^In]In-AC12-DOTA at 24 h p.i.

f Data for the gastrointestinal (GI) tract with content and carcass are presented as %ID per whole sample. The ANOVA test (Bonferroni’s multiple comparisons test) was used to test significant (*p* < 0.05) differences.

Tumor-to-organ ratios of ^111^In-labeled Affibody molecules in tumor-bearing mice 4 and 24 h after injection are presented in Table 6. There was significantly (*p* < 0.05) higher tumor-to-blood ratios for [^111^In]In-SYNT179-DOTA at 4 (31.09 ± 2.9) and 24 h (43.18 ± 13.82) p.i. compared with those for [^111^In]In-AC12-DOTA (20.60 ± 7.10 and 20.41 ± 6.75 at 4 and 24 h p.i., respectively). [^111^In]In-SYNT179-DOTA showed the highest tumor-to-organ ratios, e.g., the tumor-to-liver ratio at 4 h after injection (3.40 ± 0.49), which was significantly (*p* < 0.05) higher than that for [^111^In]In-AC12-DOTA (0.28 ± 0.05) but decreased to 0.97 ± 0.23 and 0.22 ± 0.04 at 24 h after injection for [^111^In]In-SYNT179-DOTA and [^111^In]In-AC12-DOTA, respectively. [^111^In]In-SYNT179-DOTA showed the highest tumor-to-bone ratio at 4 h after injection (168.57 ± 29.09), which was significantly higher than such ratio for [^111^In]In-AC12-DOTA (100.00 ± 29.43). This shows that [^111^In]In-SYNT179-DOTA is more favorable for imaging of bone and liver metastases at an early time point of SPECT imaging.

**Table 6:** Tumor-to-Organ Ratios for ^111^In-Labeled Affibody Molecules in BALB/C nu/nu Mice Bearing SKOV-3 Xenografts 4 and 24 h after Injection

	tumor-to-organ ratios			
	[^111^In]In-SYNT179-DOTA		[^111^In]In-AC12-DOTA	
site	4 h	24 h	4 h	24 h
blood	31.09 ± 2.9^c^	43.18 ± 13.82^d^	20.60 ± 7.10^c^	20.41 ± 6.75^d^
salivary glands	13.40 ± 2.87^a^	3.45 ± 0.89^a^	9.34 ± 2.53^b^	3.40 ± 1.13^b^
lung	10.16 ± 0.60^a^^,^^c^	4.01 ± 0.87^a^	7.38 ± 1.50^b^^,^^c^	4.05 ± 0.66^b^
liver	3.40 ± 0.49^a^^,^^c^	0.97 ± 0.23^a^^,^^d^	0.28 ± 0.05^c^	0.22 ± 0.04^d^
spleen	9.65 ± 1.56^a^^,^^c^	2.18 ± 0.68^a^^,^^d^	1.24 ± 0.43^b^^,^^c^	0.36 ± 0.09^b^^,^^d^
pancreas	17.95 ± 3.97^a^	6.77 ± 1.77^a^	12.53 ± 2.98^b^	6.26 ± 1.48^b^
stomach	13.92 ± 1.89^a^^,^^c^	5.00 ± 1.48^a^	8.45 ± 2.06^b^^,^^c^	3.97 ± 1.30^b^
small intestine	12.59 ± 1.49^a^^,^^c^	3.81 ± 0.89^a^	7.51 ± 2.14^b^^,^^c^	3.29 ± 0.54^b^
kidney	0.011 ± 0.001^a^^,^^c^	0.003 ± 0.001^a^^,^^d^	0.006 ± 0.002^b^^,^^c^	0.002 ± 0.000^b^^,^^d^
muscle	101.78 ± 46.38^a^	26.40 ± 10.03^a^	60.94 ± 15.80^b^	26.94 ± 5.28^b^
bone	168.57 ± 29.09^a^^,^^c^	57.74 ± 15.24^a^	100.00 ± 29.43^b^^,^^c^	40.58 ± 7.87^b^

a Significant difference (*p* < 0.05) between [^111^In]In-SYNT179-DOTA at 4 and 24 h p.i.

b Significant difference (p < 0.05) between[^111^In]In-AC12-DOTA at 4 and 24 h p.i.

c Significant difference (p < 0.05) between [^111^In]In-SYNT179-DOTA and [^111^In]In-AC12- DOTA at 4 h p.i.

d Significant difference (*p* < 0.05) between [^111^In]In-SYNT179-DOTA and [^111^In]In-AC12- DOTA at 24 h p.i. The ANOVA test (Bonferroni’s multiple comparisons test) was performed to test significant (*p* < 0.05) differences.

The results of head-to-head comparative biodistribution measurements of ^111^In- and ^99m^Tc-labeled Affibody molecules in mice 4 h after injection are presented in Table 7. Both ^99m^Tc-labeled conjugates demonstrated significantly (*p* < 0.05) lower renal uptake than their ^111^In-labeled counterparts. The difference was conspicuous, more than 30-fold. The liver uptake of both ^111^In-labeled constructs was 2.3–2.8-fold higher than the liver uptake of ^99m^Tc-labeled analogues (*p* < 0.05). At the same time, the accumulation of ^99m^Tc-labeled Affibody molecules in the gastrointestinal tract with the content was significantly (*p* < 0.05) higher. Taking into account that accumulation in the intestine walls was similar for all constructs, this suggests that the larger fractions of [^99m^Tc]Tc-AC12-GGGC and [^99m^Tc]Tc-SYNT179 or their radiometabolites are excreted via the bile. In the case of SYNT179, the difference between ^99m^Tc and ^111^In-labels was 4-fold. Conjugates with ^111^In-label also had significantly higher uptake (*p* < 0.05) in the lung. The activity concentration in blood was similar except for [^99m^Tc]Tc-AC12-GGGC, which demonstrated significantly higher blood retention. Although there was no difference between uptakes in bones and muscles, it was clear that the label character has an apparent effect on the uptake in the majority of normal organs and tissues. The uptake in tumor was significantly (*p* < 0.05) higher for both ^111^In-labeled Affibody molecules compared with corresponding ^99m^Tc-labeled conjugates. [^111^In]In-SYNT179-DOTA (3.63 ± 0.31%ID/g) had the highest tumor uptake.

**Table 7:** Comparative Biodistribution of ^111^In- and ^99m^Tc-Labeled Affibody Molecules in BALB/C nu/nu Mice Bearing SKOV-3 Xenografts 4 h p.i.

	uptake, %ID/g			
site	[^111^In]In-SYNT179-DOTA	[^111^In]In-AC12- DOTA	[^99m^Tc]Tc-SYNT179	[^99m^Tc]Tc-AC12-GGGC
blood	0.12 ± 0.01^c^	0.09 ± 0.02	0.11 ± 0.02^f^	0.21 ± 0.03^c^^,^^f^
salivary gland	0.28 ± 0.05	0.20 ± 0.07^e^	0.21 ± 0.03	0.62 ± 0.19^e^
lung	0.36 ± 0.02^b^	0.25 ± 0.05	0.19 ± 0.04^b^	0.29 ± 0.04
liver	1.07 ± 0.08^a^^,^^b^^,^^c^	6.43 ± 1.05^a^^,^^d^^,^^e^	0.39 ± 0.04^b^^,^^d^	2.70 ± 0.30^e^^,^^c^
spleen	0.38 ± 0.04^a^	1.49 ± 0.24^a^^,^^d^	0.15 ± 0.07^d^	0.80 ± 0.47
pancreas	0.21 ± 0.04	0.15 ± 0.05	0.09 ± 0.06	0.15 ± 0.05
stomach	0.26 ± 0.03	0.21 ± 0.02	0.28 ± 0.07	0.56 ± 0.18
small intestine	0.29 ± 0.03	0.24 ± 0.03	0.22 ± 0.06	0.31 ± 0.05
kidney	317.06 ± 8.16^b^^,^^c^	308.27 ± 30.02^d^^,^^e^	10.89 ± 3.03^b^^,^^d^	15.99 ± 3.59^c^^,^^e^
tumor	3.63 ± 0.31^a^^,^^b^^,^^c^^,^^f^	1.80 ± 0.49^a^^,^^e^	1.59 ± 0.19^b^^,^^f^	0.84 ± 0.18^c^^,^^e^^,^^f^
muscle	0.04 ± 0.01	0.03 ± 0.01	0.011 ± 0.002	0.06 ± 0.05
bone	0.02 ± 0.01	0.018 ± 0.001	0.008 ± 0.003	0.03 ± 0.01
low GI tract^g^	0.54 ± 0.15	0.84 ± 0.24	2.18 ± 0.90	2.19 ± 0.37
carcass^g^	3.22 ± 0.07	2.59 ± 0.39	1.56 ± 0.68	4.17 ± 0.70

a Significant difference (*p* < 0.05) between [^111^In]In-SYNT179-DOTA and [^111^In]In-AC12- DOTA.

b Significant difference (p < 0.05) between [^111^In]In-SYNT179-DOTA and [^99m^Tc]Tc-SYNT179.

c Significant difference (p < 0.05) between [^111^In]In-SYNT179-DOTA and [^99m^Tc]Tc-AC12-GGGC.

d Significant difference (p < 0.05) between [^111^In]In-AC12-DOTA and [^99m^Tc]Tc-SYNT179.

e Significant difference (p < 0.05) between [^111^In]In-AC12-DOTA and [^99m^Tc]Tc-AC12-GGGC.

f Significant difference (p < 0.05) between[^99m^Tc]Tc-SYNT179 and [^99m^Tc]Tc-AC12-GGGC. The ANOVA test (Bonferroni’s multiple comparisons test) was performed to test significant (*p* < 0.05) differences.

g Data for the gastrointestinal (GI) tract with content and carcass are presented as %ID per whole sample.

Data concerning the comparison of tumor-to-organ ratios are presented in Table 8. The tumor-to-blood ratios for [^111^In]In-SYNT179-DOTA and [^111^In]In-AC12-DOTA were significantly (*p* < 0.05) higher compared with those for [^99m^Tc]Tc-SYNT179 and [^99m^Tc]Tc-AC12-GGGC, respectively. There were no significant differences between tumor-to-liver ratios for [^111^In]In-SYNT179-DOTA and [^99m^Tc]Tc-SYNT179 or between [^111^In]In-AC12-DOTA and [^99m^Tc]Tc-AC12-GGGC. However, these ratios were significantly (*p* < 0.05) higher for both ^111^In- and ^99m^Tc-labeled derivatives of SYNT179 than for derivatives of AC12 labeled with the same nuclides. In the case of SYNT179, the label character had no impact on the tumor-to-bone, tumor-to-lung, and tumor-to-muscle ratios (no significant difference). However, the ^111^In-DOTA label provided 2–3-fold higher ratios in these organs for AC12 than ^99m^Tc-GGCC.

**Table 8:** Tumor-to-Organ Ratios for ^111^In- and ^99m^Tc-Labeled Affibody Molecules in BALB/C nu/nu Mice Bearing SKOV-3 Xenografts 4 h p.i

	tumor-to-organ ratios			
site	[^111^In]In-SYNT179-DOTA	[^111^In]In-AC12-DOTA	[^99m^Tc]Tc-SYNT179	[^99m^Tc]Tc-AC12-GGGC
blood	31.09 ± 2.91^b^^,^^c^	20.60 ± 7.10^e^	15.74 ± 4.13^b^^,^^f^	4.11 ± 0.87^c^^,^^e^^,^^f^
salivary gland	13.40 ± 2.87^c^	9.34 ± 2.53^e^	7.61 ± 1.36^f^	1.39 ± 0.22^c^^,^^e^^,^^f^
lung	10.16 ± 0.60^c^	7.38 ± 1.50	8.63 ± 1.95	2.90 ± 0.32^c^
liver	3.40 ± 0.49^a^^,^^c^	0.28 ± 0.05^a^^,^^d^	4.17 ± 0.60^d^^,^^f^	0.34 ± 0.03^c^^,^^f^
spleen	9.65 ± 1.56^a^^,^^c^	1.24 ± 0.43^a^^,^^d^	13.11 ± 7.79^d^^,^^f^	0.90 ± 0.14^c^^,^^f^
pancreas	17.95 ± 3.97^c^	12.53 ± 2.98	20.11 ± 7.33^f^	5.83 ± 0.62^c^^,^^f^
stomach	13.92 ± 1.89^c^	8.45 ± 2.06^e^	6.04 ± 1.89	1.53 ± 0.23^c^^,^^e^
small intestine	12.59 ± 1.49^c^	7.51 ± 2.14	7.41 ± 1.49^f^	2.67 ± 0.38^c^^,^^f^
kidney	0.011 ± 0.001^c^	0.006 ± 0.002^d^^,^^e^	0.156 ± 0.047^d^	0.05 ± 0.01^c^^,^^e^
muscle	101.78 ± 46.38	60.94 ± 15.80^d^^,^^e^	156.22 ± 38.60^d^^,^^f^	22.78 ± 3.83^e^^,^^f^
bone	168.57 ± 29.09^a^^,^^c^	100.00 ± 29.43^a^	208.04 ± 69.05	35.01 ± 12.77^c^

a Significant difference (*p* < 0.05) between [^111^In]In-SYNT179-DOTA and [^111^In]In-AC12- DOTA.

b Significant difference (p < 0.05) between [^111^In]In-SYNT179-DOTA and [^99m^Tc]Tc-SYNT179.

c Significant difference (p < 0.05) between [^111^In]In-SYNT179-DOTA and [^99m^Tc]Tc-AC12-GGGC.

d Significant difference (p < 0.05) between [^111^In]In-AC12-DOTA and [^99m^Tc]Tc-SYNT179.

e Significant difference (p < 0.05) betweenll [^111^In]In-AC12-DOTA and [^99m^Tc]Tc-AC12-GGGC.

f Significant difference (p < 0.05) between [^99m^Tc]Tc-SYNT179 and [^99m^Tc]Tc-AC12-GGGC. The ANOVA test (Bonferroni’s multiple comparisons test) was performed to test significant (*p* < 0.05) differences.

**Figure 7:**
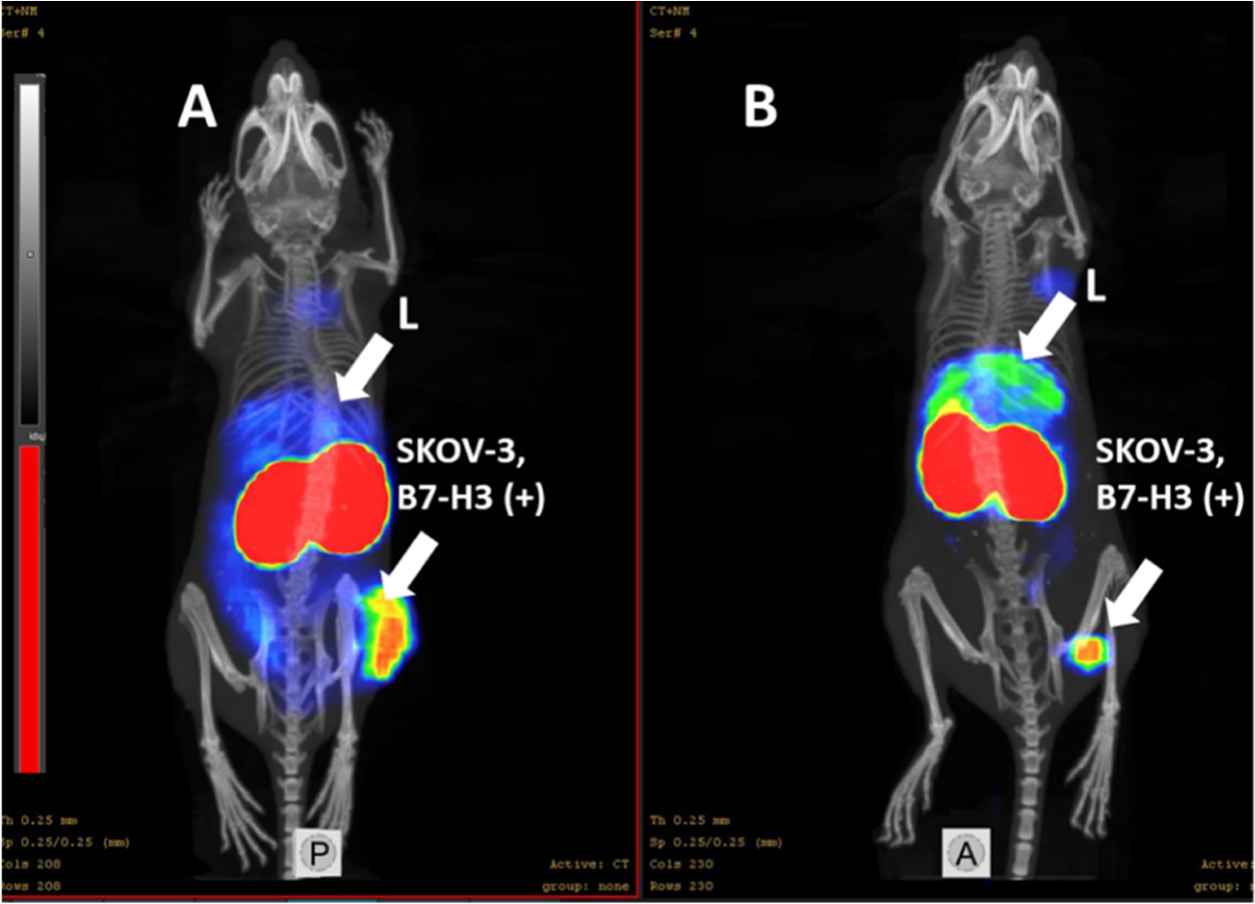
Imaging of (A) [^111^In]In-SYNT179-DOTA and (B) [^111^In]In-AC12-DOTA in BALB/C nu/nu mice bearing B7–H3-positive SKOV-3 xenografts 4 h p.i. 3 μg of labeled Affibody molecules (3 MBq, 100 μL in PBS) were injected into the tail vein. A linear relative scale (arbitrary units normalized to a maximum count rate) is provided for the image. The scale was adjusted to show red pixels in xenografts. Arrows point at the tumor (T) and liver (L).

**Figure 8:**
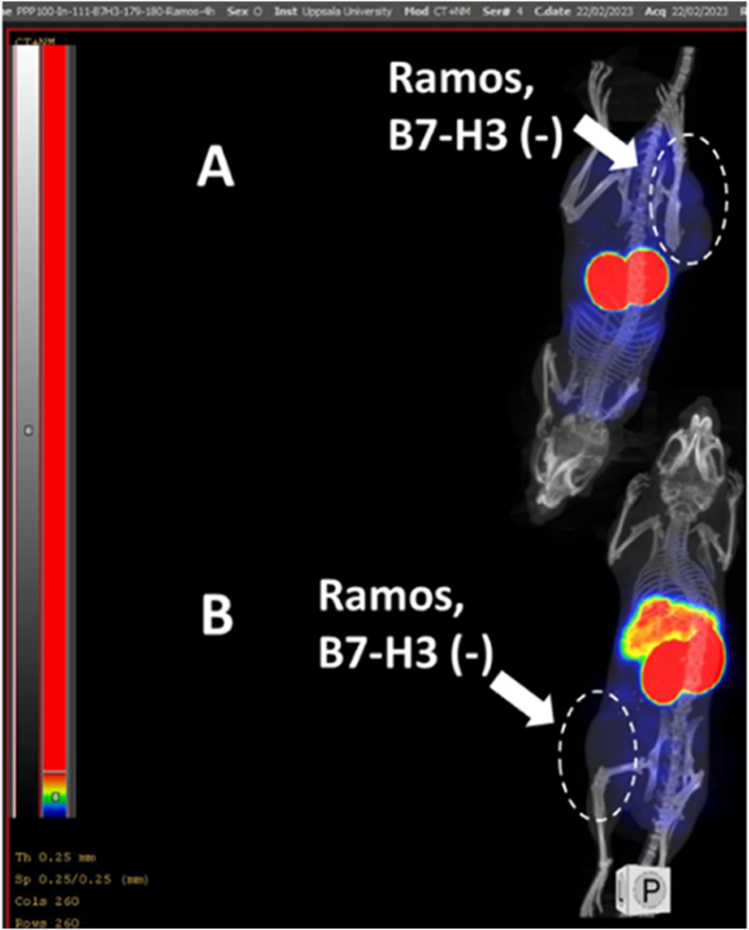
Imaging of (A) [^111^In]In-SYNT179-DOTA and (B) [^111^In]In-AC12-DOTA in BALB/C nu/nu mice bearing B7–H3-negative Ramos xenografts 4 h p.i. The labeled Affibody molecule (3 μg, 1 MBq in 100 μL in PBS) was injected into the tail vein.

The nanoSPECT/CT imaging (Figure 7) of [^111^In]In-SYNT179-DOTA and [^111^In]In-AC12-DOTA in BALB/C nu/nu mice bearing B7–H3-positive SKOV-3 xenografts 4 h after injection confirmed ex vivo biodistribution. A pronouncedly less accumulation of activity in the liver and higher accumulation in the tumor for [^111^In]In-SYNT179-DOTA compared to [^111^In]In-AC12-DOTA was observed. Activity uptake in B7–H3-negative Ramos xenografts was appreciably lower than that in the B7–H3-positive SKOV-3 xenograft, confirming target-specific binding of the ^111^In-labeled conjugates in vivo (Figures 7 and 8).

## 2 Discussion

For the successful clinical application of scaffold proteins, it is essential to maximize radionuclide delivery and retention in tumors while minimizing their accumulation in nontarget organs and tissues. For imaging applications, this precondition provides high imaging contrast and, therefore, high sensitivity. The main focus of this study is to evaluate the effect of the labeling strategy on the imaging properties of B7–H3-targeting Affibody molecules with the ultimate goal of improving quantitative visualization of B7–H3-expressing tumors. The feasibility of visualizing B7–H3-expressing tumors in vivo using [^99m^Tc]Tc-AC12-GGGC Affibody molecules was shown earlier.^19^ However, the tumor uptake was suboptimal, and the targeting properties of the Affibody molecules needed to be improved to obtain a higher signal from the tumor site and less background uptake, resulting in a higher imaging contrast. An improvement in the binding affinity of anti-B7–H3 Affibody molecules by affinity maturation resulted in an appreciably higher imaging contrast.^26^ It should be noted that once a novel targeting probe is introduced, further optimization is generally required. Previous studies have demonstrated that the selection of the appropriate labeling chemistry is critical.^16^ Different combinations of a radionuclide and chelator used for coupling to Affibody molecules modify the physicochemical properties of the water-exposed surface of Affibody molecules in different ways. Such modifications might affect the affinity of their binding to molecular targets, a weak binding to blood proteins, off-target interactions with normal tissues, and the predominant way of excretion.^16^

Thus, we aimed to test the targeting properties of Affibody molecules labeled with ^111^In. This radionuclide is typically conjugated to proteins via aminopolycarboxylate chelators, which increases the hydrophilicity of proteins. Physical properties of ^111^In (*T*_1/2_ = 2.83 days), Eγ = 173 keV (90.5%) and 247 keV (94%) make it a suitable radioisotope for radionuclide molecular imaging using single-photon emission computed tomography (SPECT). For labeling with ^111^In, the DOTA chelator was coupled to Affibody molecules. This chelator provides exquisite stable labels in vivo due to the kinetic inertness of chelates.^28^ Importantly, Affibody molecules can withstand the elevated temperatures required for stable chelation via DOTA. ^111^In as a typical residualizing trivalent metal, and the major features of biodistribution of ^111^In might be generalized (with some reservations) for analogues labeled with other trivalent radiometals, such as positron emitters ^68^Ga, ^66^Ga, or ^86^Y. The long half-life of ^111^In permits us to explore if the time extension between injection and imaging would increase an imaging contrast due to quicker washout of the radionuclide from normal tissues than from tumors. Labeling with ^111^In was performed with high efficiency (Table 2), generating highly stable conjugates (Table 3). The binding of ^111^In-labeled constructs to two B7–H3-expressing cell lines was highly B7–H3-specific in vitro (Figure 4). Affinity measurement showed that the use of the ^111^In-label somewhat increased the strength of binding to living B7–H3-expressing cells for AC12 compared with the use of ^99m^Tc (Table 4), but for SYNT179, the pattern was the opposite. Thus, the labeling had an impact on the binding strength of the Affibody molecules. The cellular processing (Figure 5) showed that the total cell-associated activity of [^111^In]In-SYNT179-DOTA on SKOV-3 was 2.7-fold higher than that for [^111^In]In-AC12-DOTA after 24 h of incubation. The internalization was slow, which is typical for Affibody-based probes.

Biodistribution data in tumor-bearing mice 4 and 24 h after injection (Table 3) demonstrated that the tumor uptake of [^111^In]In-SYNT179-DOTA was 2-fold higher than that for [^111^In]In-AC12-DOTA at both time points. The higher affinity of [^111^In]In-SYNT179-DOTA could be an apparent reason for the higher tumor uptake. The uptake of both [^111^In]In-SYNT179-DOTA and [^111^In]In-AC12-DOTA in B7–H3-positive SKOV-3 xenografts was significantly higher than that in B7–H3-negative Ramos xenografts (Figure 6). This indicated that tumor accumulation was B7–H3-mediated in vivo. The uptake of both ^111^In-labeled Affibody molecules in normal tissues decreased only less than 2-fold between 4 and 24 h after injection. The reduction of renal uptake was in the same range as the reduction in other organs and tissues. This indicates that the tracers are internalized in normal tissues, and the residualizing properties of the [^111^In]In-DOTA label are crucial for their retention there. The tumor uptake of both variants decreased more than 4-fold by 24 h compared to 4 h. This suggests that both variants are not internalized after binding to B7–H3 in vivo (as was predictable from our in vitro studies) and can dissociate and be washed out. Thus, the increase in affinity for [^111^In]In-SYNT179-DOTA might not be sufficient to completely prevent dissociation from the target in vivo and its release from tumors when the expression is in such a low level. These processes resulted in the decrease of the tumor-to-organ ratios 24 h after injection. Thus, the use of long-lived radionuclides does not improve the contrast of imaging using derivatives of SYNT179-DOTA and AC12-DOTA.

The binding of a radiolabeled peptide to blood proteins might slow its clearance rate and create an elevated background.^29^ The binding correlates with lipophilicity but is also dependent on an interplay of each amino acid residue with its environment.^30,31^ Selection of an optimal labeling approach might suppress the negative effect of binding to the blood proteins.^29^ This study presents another example. We have found earlier that the blood retention of [^99m^Tc]Tc-AC12-GGGC is twice higher than the retention of [^99m^Tc]Tc-SYNT179.^19,26^ Coupling of a hydrophilic ^111^In-DOTA compensated for this, and the blood concentration of [^111^In]In-AC12-DOTA was on the same level (no significant difference, *p* > 0.05) as the blood concentration of [^99m^Tc]Tc-SYNT179 at the same time point (Table 7). A radiolabel’s physicochemical properties influence the intracellular retention of a radionuclide. This might affect not only the uptake of a tracer in tumors but also the uptake in normal tissues, where the construct is internalized. In this study (Table 7), this effect was the most pronounced in kidneys, where ^111^In retained to a much higher extent (30-fold compared to ^99m^Tc-labeled counterparts) and in the liver (2-fold higher uptake of ^111^In-labeled variants). High renal uptake is a feature of many scaffold proteins.^32−34^ The use of conventional approaches to the reduction of renal uptake results in very limited success. The renal reabsorption mechanism is unclear, but megalin is not involved.^35^ The high renal reabsorption does not preclude the use of Affibody molecules for molecular imaging (renal metastases are seldom). HER2-binding Affibody visualized renal metastases on the top of the kidney.^36^ The retention of ^111^In in the lung was also higher than the retention of ^99m^Tc delivered with a homologous targeting Affibody molecule. The hepatic uptake was dependent on the composition of Affibody molecules. Thus, the liver uptake of AC12 variants was appreciably higher than the uptake of SYNT179 for both ^111^In and ^99m^Tc labels. The use of the more hydrophilic [^111^In]In-DOTA label resulted in a lower percentage of activity excreted via bile into the gastrointestinal tract, creating a better precondition for imaging of abdominal metastases. It has to be noted that there was no effect of the label on the uptake in the pancreas, stomach, intestines wall, muscle, or bone.

The tumor uptake in the case of ^111^In-labeled Affibody molecules was significantly higher than the uptake of ^99m^Tc-labeled counterparts. The internalization rates of both [^111^In]In-SYNT179-DOTA and [^111^In]In-AC12-DOTA are low, and although even a minor internalized fraction will result in a marked difference in retention, the high tumor uptake of ^111^In-labeled Affibody molecules may not be fully explained by the residualizing properties of the [^111^In]In-DOTA label. More research is needed to understand this.

From an imaging point of view, the most important is the contrast with the organs, which frequently harbor metastases of different malignancies, such as liver, lung, and bones. The tumor-to-blood ratio is also a critical parameter, since the activity in the bloodstream reduces the contrast. The higher tumor uptake of ^111^In-labeled variants counteracted their elevated uptake in the liver and lung, and there were no differences in tumor-to-liver and tumor-to-lung ratios between the most promising variant, [^111^In]In-SYNT179-DOTA, and its ^99m^Tc-labeled analogue, [^99m^Tc]Tc-SYNT179. In the case of AC12 derivatives, tumor-to-lung, tumor-to-stomach, and tumor-to-muscle ratios were higher in the case of the ^111^In-label. However, this variant was clearly inferior to [^111^In]In-SYNT179-DOTA. The most important tumor-to-blood ratio was the highest for [^111^In]In-SYNT179-DOTA; thus, this variant was clearly superior. The high kidney uptake would not permit the use of this tracer in primary renal cell carcinomas.

As pointed out above, the optimal imaging time using SYNT179-DOTA should be within 3–4 h after injection. This indicates that more short-lived radionuclides than ^111^In might suit better. One obvious candidate is ^68^Ga, which is a positron emitter permitting the advantage of PET in radionuclide imaging.

## 3 Conclusions

In this study, radiolabeling of anti-B7–H3 Affibody molecules with ^111^In resulted in a residualizing label with an improved tumor uptake and a significantly higher tumor-to-blood ratio (less background signal) compared to nonresidualizing ^99m^Tc-labeled Affibody molecules. SYNT179 labeled with ^111^In could be a promising imaging agent for preclinical visualization B7–H3-expressing tumors at the day of administration.

## 4 Methods

### 4.1 General

[^111^In]InCl_3_ was purchased from Curium Pharma, Netherlands, V., Petten, The Netherlands for labeling of Affibody molecules with ^111^In. ^99m^Tc as pertechnetate by elution of an Ultra TechneKow generator (Mallinckrodt, Petten, The Netherlands) with sterile 0.9% sodium chloride (Mallinckrodt, Petten, The Netherlands) was used. The iTLC analysis was performed using a CR35 BIO Plus Storage Phosphor System and AIDA image analysis software (from ElysiaRaytest, Bietigheim-Bissingen, Germany). An automated γ-spectrometer with a 3-inch NaI (TI) well detector (2480 Wizard, Wallac, Turku, Finland) was used to measure activity from cell and animal samples. For labeling and injection formulation, radioactivity was measured using a dose calibrator RDC-VIK-202 (COMECER Netherlands, 8501-XC, Jourse, Netherlands) equipped with an ionizing chamber.

B7–H3-expressing ovarian cancer SKOV-3 and breast cancer BT-474 cell lines, from the American Type Culture Collection (ATCC), were used for in vitro studies. To establish B7–H3-positive and -negative xenografts, SKOV-3 and Ramos lymphoma cells (ATCC) were implanted in BALB/C nu/nu mice, respectively. Cells were cultured in RPMI medium (Flow Laboratories, Irvine, U.K.) supplemented with fetal bovine serum (10 and 20% for SKOV-3 and BT-474, respectively), 2 mM l-glutamine, 100 IU/mL penicillin, and 100 mg/mL of streptomycin.

To show significant differences (*p* < 0.05), data from in vitro studies and biodistribution were analyzed by unpaired 2-tailed *t*-test and ANOVA using GraphPad Prism (version 10.4.1 for Windows; GraphPad Software), respectively.

### 4.2 Production, Purification, and Characterization of Anti-B7–H3 Affibody Molecules

The Affibody molecules were produced by chemical synthesis and characterized as described previously.^26^ For DOTA conjugation, the synthesized Affibody molecules were reduced in 40 mM DTT for 1 h at 30 °C, followed by buffer exchange to conjugation buffer (0.2 M NaAc, pH 5.6, treated with Chelex 100 resin), using a PD-10 desalting column. In brief, the PD-10 column was equilibrated with 5 × 5 mL of conjugation buffer prior to loading a 1.5 mL sample, followed by 1 mL of conjugation buffer before elution with 3 mL of conjugation buffer. The concentration of the eluted sample was measured by absorbance readings at 280 nm using a NanoDrop 8000 Spectrophotometer. Maleimido-monoamide-DOTA (Macrocyclics) was added at 3.4-fold molar excess, and the samples were incubated at 22 °C and 600 rpm for 60 min. The buffer was exchanged with 0.2 M NaAc, pH 5.6 (treated with Chelex 100 resin) using a PD-10 desalting column as described above. The conjugated Affibody molecules were analyzed by RP-HPLC-MS using an Agilent 1290 Infinity UHPLC- system, equipped with a single quadrupole and AP-ESI and the analytical column Zorbax 300SB-C8 RRHD (Agilent).

### 4.3 Radiolabeling and In Vitro Stability

For labeling with ^111^In, SYNT179-DOTA and AC12-DOTA Affibody molecules (40 μg, 22 μL, dissolved in 0.2 M NH_4_Ac, pH 5.5) were incubated with [^111^In]InCl_3_ (20–40 MBq, 20–40 μL of the stock) at 90 °C for 60 min. Loosely bound ^111^In was removed using incubation of the reaction mixture with a 500-fold molar excess of ethylenediaminetetraacetic acid tetrasodium salt (11 μL of 100 mg/mL EDTANa_4_ dissolved in 0.2 M NH_4_Ac, pH 5.5) at 90 °C for 10 min. Radiochemical yield was analyzed using instant thin layer chromatography (iTLC, Agilent Technologies, Santa Clara, CA) developed with 0.2 M citrate buffer pH 2. A NAP-5 column, pre-equilibrated and eluted with 1% BSA in PBS, was used for purification. Radiochemical purity was analyzed the same as radiochemical yield.

To cross-validate radio-iTLC data further, reversed-phase HPLC was conducted using an EliteLaChrom system (Hitachi, VWR, Darmstadt, Germany) consisting of an L-2130 pump, a UV detector (L-2400), and a radiation flow detector (Bioscan, Washington, DC) coupled in series. A purity analysis was performed using an analytical column (Vydac RP C18 column, 300 Å; 3 mm × 150 mm; 5 μm). HPLC conditions were as follows: *A* = 10 mM TFA/H_2_O, *B* = 10 mM TFA/acetonitrile, UV-detection at 280 nm, gradient elution: 0–15 min at 5–70% B, 15–18 min at 70–95% B, 19–20 min at 5% B, and a flow rate of 1.0 mL/min.

Labeling of control constructs SYNT179 and AC12-GGGC with ^99m^Tc using a lyophilized kit was performed as descried earlier.^37^ The radiochemical yield was >95% for both radioconjugates. Therefore, no further purification was performed.

Freshly purified fractions of the radiolabeled conjugates (10 μL, 1 μg) were incubated with PBS for 1, 4, and 24 h at 37 °C to evaluate in vitro stability. The test was run in triplicates.

### 4.4 In Vitro Studies

B7–H3 expression levels were estimated to be 68,000, 45,000, and 250 receptors per cell for SKOV-3, BT-474, and Ramos, respectively.^19^ B7–H3-expressing cells (SKOV-3 and BT-474) were seeded in cell culture dishes (35 mm in diameter) with a density of 10^6^ cells/dish for an in vitro study. A set of three dishes was used for in vitro studies.

The binding specificity of radiolabeled conjugates to living B7–H3-expressing cells was tested using a saturation experiment, as described earlier.^19^ 200-fold excess of nonlabeled Affibody molecules and 10 nM of the ^111^In-labeled Affibody molecules were used for this experiment.

The affinity of binding of ^111^In- labeled Affibody molecules, the kinetics of binding, and their dissociation from living SKOV-3 cells were measured using a LigandTracer Yellow instrument (Ridgeview Instruments AB, Vänge, Sweden), as previously described.^38,39^ Uptake curves were recorded at 1, 3, and 9 nM for [^111^In]In-SYNT179-DOTA. 2, 6, and 18 nM were used for [^111^In]In-AC12-DOTA.

Cellular processing of ^111^In-labeled Affibody molecules during continuous incubation was studied as previously described.^40^ Radiolabeled Affibody molecules (10 nM) were added to the cells and incubated at 37 °C in a humidified incubator for 1, 2, 4, 8, and 24 h.

### 4.5 In Vivo Studies

Animal experiments were performed according to the national legislation for work with laboratory animals granted by the Ethical Committee for Animal Research in Uppsala (permit 5.8.18–00473/2021, approved 26 February 2021).

Biodistribution of ^111^In-labeled Affibody conjugates was studied in BALB/C nu/nu mice bearing B7–H3-positive SKOV-3 xenografts (10^7^ cells/mouse were subcutaneously injected on the right hind leg of female BALB/c nu/nu mice). For in vivo specificity, B7–H3-negative Ramos cells (6 × 10^6^ cells/mouse) were subcutaneously implanted on the left hind leg of mice. The experiments were performed 3 weeks after cell implantation. Four mice per data point were used for the animal experiments. The average animal weight was 17.8 ± 1.3 g. The average tumor weights were 0.10 ± 0.06 and 0.7 ± 0.4 g for SKOV-3 and Ramos xenografts, respectively. Biodistribution of ^111^In-labeled Affibody molecules was measured 4 and 24 h after injection in mice bearing SKOV-3 xenografts. Groups of four tumor-bearing mice were injected with ^111^In-labeled Affibody molecules (0.4 nmol, 20 kBq, 100 μL in PBS) into the tail vein. To test B7–H3-specific accumulation, one group of animals bearing B7–H3-negative Ramos xenografts was injected with the same peptide and activity doses for each conjugate, and the biodistribution was measured 4 h after injection. Mice were euthanized by overdosing of anesthetic solution (20 μL of solution per gram of body weight: ketamine, 10 mg/mL; xylazine, 1 mg/mL) followed by a heart puncture. Blood, organ, and tissue samples were collected and weighed. Radioactivity was measured using a γ counter along with three standards and empty syringes for each animal. Uptake values for each organ/tissue were calculated as the percentage of the injected dose per gram of tissue (%ID/g). For comparison, two groups of mice were injected with [^99m^Tc]Tc-SYNT179 and [^99m^Tc]Tc-AC12-GGGC (0.4 nmol, 60 kBq, 100 μL in PBS), and the biodistribution measurements were performed at 4 h after injection as described above.

To confirm biodistribution results, small animal SPECT/CT imaging was performed. One SKOV-3-bearing mouse was intravenously injected with each ^111^In-labeled Affibody molecule (0.4 nmol, 2 MBq). To confirm in vivo specificity, one mouse bearing Ramos xenograft was intravenously injected with the same peptide and activity dose for each ^111^In-labeled Affibody molecule. The mice were scanned at 4 h after injection using a nano SPECT/CT scanner (Mediso Medical Imaging Systems, Budapest, Hungary). The mice were euthanized by CO_2_ asphyxiation immediately before being placed in the camera. The computed tomography (CT) acquisition was carried out at the following parameters: energy peak of 50 kV, 670 μA, 480 projections, and 2.29 min acquisition time. SPECT acquisition was performed at the following parameters: window width of 20%, matrix of 256 × 256, and acquisition time of 1 h. CT images were reconstructed in real time using Nucline 2.03 Software (Mediso Medical Imaging Systems, Budapest, Hungary). SPECT raw data were reconstructed by using TeraTomo 3D SPECT reconstruction technology. SPECT and CT files were fused using Nucline 2.03 Software and are presented as maximum intensity projections on an RGB color scale.

## Abbreviations Used

HER2: human epidermal growth factor receptor 2
RP-HPLC-MS: reverse-phase high-performance liquid chromatography-mass spectrometry
kDa: kilodalton
PBS: phosphate-buffered saline
*T*_1/2_: half-life
p.i.: postinjection
